# Assessment of High Reliability Organizations Model in Farabi Eye Hospital, Tehran, Iran

**Published:** 2018-01

**Authors:** Seyed Mohammad Hadi MOUSAVI, Mahmoud JABBARVAND BEHROUZ, Hojjat ZERATI, Hossein DARGAHI, Akram ASADOLLAHI, Seyed Ahmad MOUSAVI, Elham ASHRAFI, Abolfazl ALIYARI

**Affiliations:** 1. Health Information Management Research Center, Tehran University of Medical Sciences, Tehran, Iran; 2. Eye Research Center (ERC), Farabi Eye Hospital, Tehran University of Medical Sciences, Tehran, Iran; 3. Dept. of Epidemiology and Biostatistics, School of Public Health, Tehran University of Medical Sciences, Tehran, Iran; 4. Deputy of Planning and Development of Resources, Tehran University of Medical Sciences, Tehran, Iran

**Keywords:** High reliability organizations model, Knowledge, Staffs and managers, Iran

## Abstract

**Background:**

A high-reliability organization (HRO) is a separate paradigm can indicate medical error reduction and patient safety improvement. Hospitals, as vital organizations in the health care system, can transform to HROs to achieve optimal performance and maximum safety in order to manage unpredicted events efficiently. Therefore, the aim of this research was to determine the knowledge of managers and staffs of Farabi Eye Hospital, Tehran, Iran about HROs model, and the extent of HROs establishment in this hospital in 2015–2016.

**Methods:**

In this descriptive-analytical and cross-sectional study, data were collected through HROs questionnaire and checklist. Validity of questionnaire and checklist was confirmed by expert panel, and the questionnaire reliability by Alpha-Cronbach method with 0.85. The collected data were analyzed with Spearman’s correlation coefficient and Mann-Whitney test using the SPSS software version 19.

**Results:**

Most of the respondents were familiar with HROs model to some extent and only 18.8% had a high level of knowledge in this regard. In addition, there was no significant correlation between the knowledge of staffs and managers with establishment of HROs model in Farabi eye hospital.

**Conclusion:**

Managers and staffs of Farabi Eye Hospital did not have a high knowledge level of the model of HROs and had little information about the functions and characteristics of these organizations. Therefore, we suggest HROs training courses and workshops should be established in this hospital to increase the knowledge of the managers and staffs for better establishment of HROs model.

## Introduction

Achieving high reliability should be the main goal of the hospitals as part of the health care organizations. They should make effort to accomplish this organizational goal, and employ effective and efficient management in this regard. Nowadays, considering the necessity of the improvement of quality and safety in hospitals, new ways should be constantly sought to enhance these two factors in hospitals and the other health care organizations (1, 2).

The establishment of a health care system with a safe structure requires a separate paradigm known as “high-reliability organizations (HROs)”, which indicates medical error reduction and patient safety improvement in health care organizations. In other words, the structure and managing of a heath care organization can decrease the occurrence of medical error, and improve organizational safety accordingly (3).

By definition, HROs are organizations managed to avoid environmental crises through their activities, although accidents and incidents may naturally occur in these organizations due to complex and high-risk factors. HROs are organizations with complex and risky, yet safe and effective characteristics and features (4). The main elements of these organizations are commitment to the observation of safety regulations and development of a safety and learning culture (5).

HROs were first established by a group of researchers from University of California, Berkeley. The history of HROs goes back to high-risk industries, aircraft carriers, air traffic control, commercial aviation in general, military operations, and nuclear energy. They were then employed in health care systems (6).

Although HROs are different, but they are very similar to one another. The first similarity is that they all have social environments, and the second is that they all use high-risk technologies. As for the third similarity, the possible outcomes of errors and mistakes in these organizations will lead to learning through trial and error. Finally, to prevent mistakes and errors, HROs have designed and employed processes to manage complicated technologies and tasks (7). Further researches on these organizations were conducted in the commanding system of unexpected events and fire departments, and the NICU of Loma Linda University Hospital (8, 9).

Some distinct features of these organizations are a large capacity for accepting constructive criticism in the level of hospital management, periodic inspections to prevent errors and mistakes, having knowledge and positive thinking about the causes of the errors, and accountability of the managers of these organizations (10).

High reliability organizations have emphasis on the dynamic nature of trust, which means that they constantly look for ways to enhance reliability and avoid mistakes and errors, and to compensate for the errors rapidly; in other words, HROs search for credibility more than mere being preoccupied with it. HROs are not identified by their errors and mistakes; it is their effective management of high-risk technologies through monitoring and surveying the dangers and probabilities that make them trust seekers (11).

It is concluded that HROs share some distinct features such as being sensation to failure, reluctance to simplify interpretations, sensitivity to operation, commitment to resilience, and deference to expertise (12).

In other words, HROs are special organizations due to their efforts for organizing how to increase the quality of attention to the whole organization, and enhancing the knowledge of the staffs on details. Therefore, HROs seek the ways to respond to possible incidents. HROs are successful organizations that constantly correct and rebuild themselves (13).

Researchers have also identified some other common features of HROs like advanced technology, trained and eligible staffs, continuous education, effective reward system, efficient auditing of the processes and mechanisms, and continuous effort for progress (14,15). Moreover, high-quality performance, feeling of voluntary services, commitment to accountability and responsibility to create reliability, and having concerns about misunderstanding, unawareness, and wrong performance in undertaking organizational functions, and increasing the inspections as a precaution against potential threats, are among the other specifications of these organizations (16).

On the other hand, the managers and experts of the health care system look for the ways to present safer and more reliable care. In fact, from the viewpoint of continuous performance, it is very helpful to see health care organizations seek to be reliability systems and follow a series of continuous activities to institutionalize them (17). To gain the public trust, hospitals need to become trustable in terms of patient safety, delivering effective clinical care, and integration of ethical ideals expected by the public. There is no doubt that hospitals are under scrutiny by the public and any wrong performance can weaken their trust (18).

To protect the patients and staffs, the regulations of the hospitals and their governing authority and monitoring organizations have expanded like Joint Commission for Accreditation of Health Organization (JCAHO) (19). There are many national regulations about the needs of the hospitals for safety and reporting the errors that may cause damage to patients and staffs. Human errors were defined as any situation in which the planned orders and consequences of physical or mental activity fails to achieve its goals because of an inappropriate planning or unplanned performances (20).

However, many hospital incidents occur every year. A comprehensive report of a medical organization entitled “To Err IS Human” explained that 44000–98000 Americans were injured because of medical errors. Preventable complications are a major cause of mortality and morbidity in the US (21,22). High-performance reliability hospitals have 40% higher performance with fewer errors when compared to low-performance hospitals. Moreover, 70% of the reported errors are expectable, and at least about 50% of them in health care systems are not reported (23).

In most cases, physicians or nurses do not make a mistake, but human errors have roots on different factors like defective systems, inadequate education, and weak safety culture and methods (24, 25). Due to excellent safety and effectiveness records, HROs have increased substantially in the past 15 years (26,27). Therefore, many organizations try to become a high-reliability organization (28).

The aim of this study was to determine the knowledge level of the administrators and staffs of medical and non-medical departments of Farabi Eye Hospital about HROs, and the extent of HROs establishment in this hospital.

## Materials and Methods

This cross-sectional and descriptive-analytical study was conducted in Farabi Eye Hospital in Tehran, Iran in 2015–2016. The medical departments of the hospital included Emergency, ICU, angiography, radiology, optometry, stem cell bank, clinical laboratory, and outpatient clinics, and also non-medical departments of the hospital comprised storehouse, occupational health, nutrition, laundry, IT center, central sterilization, medical records statistics, public relations, excellence, patient safety, social work, supplies, library, audiovisual center, secretariat of the seminars, and medical instrument were assessed by HROs checklist.

The research tool for this research was HROs questionnaire and a checklist, respectively. The researcher-made questionnaire on knowledge about the HROs model had 24 questions used in this study after its validity was confirmed by a group of experts, and its reliability was assessed using the Cronbach’s alpha (0.85). The questionnaire was distributed to 80 administrators and staffs of the departments, and collected after completion.

With regards to observation of ethical consideration, the researchers acquired the permission of Farabi Hospital senior managers and assured the respondents, the confidentibility of information would be observed in this research.

The checklist used for the assessment of HROs establishment first was translated into Persian by a language expert. Then, its validity was confirmed after backward translation and comparison of the backward and forward translation by a panel of experts. The checklist contained five domains of the features and elements of HROs, including assessment of attention to patient safety, hospital concern about correcting medical errors, reluctance to simplify interpretations by the staffs and managers, sensitivity to operation, commitment to resilience in the organization, and the deference to expertise. The data of the checklist was collected through interviews with 80 managers and staffs of medical and non-medical departments, and witnessing the observation of HRO components by census method. The knowledge of the respondents and the HROs establishment level were determined using a 3-point scale (“not at all”, “to some extent”, and “very much”). As for scoring, a score below 50% was considered “not at all”, a score of 50%–75% was considered “to some extent”, and a score above 75% was considered “very much”. Data analysis was performed with SPSS version 16 (Chicago, IL, USA) using Spearman’s correlation coefficient and Mann-Whitney test. *P* values less than 0.05 were considered significant.

## Results

The following tables present the results of the analysis of the data obtained from 80 medical and nonmedical departments’ managers and staffs regarding their knowledge of HROs and assessment of HROs model. The results of Table 1 showed that 81.2% of the respondents were familiar with the model of HROs to some extent and 18.8% had a high level of knowledge in this regard. Moreover, the highest (41.2%) knowledge level was related to “how to prevent personal errors and mistakes” and the lowest level of knowledge was related to “important factors in the identification of HROs” as 60% of the participants declared to have no knowledge in this regard. Moreover, the knowledge level was higher for “how to gain experience from the mistakes and errors of other staffs” and “how to access to required resources to confront unexpected events” versus items such as “avoiding simplification of analysis in HROs”, “Dimensions of the model of HROs”, and “How to develop the culture of HROs”.

**Table 1:** Absolute and relative distribution of knowledge level of Farabi Hospitals’ managers and staff about HROs model

***Row***	***Dimensions of HROs questions***	***Knowledge Level***		
		**Not at all N (%)**	**To some extent N (%)**	**Very much N (%)**
1	Dimensions of model HROs	43(53.8)	36(45)	1(1.2)
2	How to develop the culture of HROs model	43(53.8)	35(43.8)	2(2.5)
3	Activities of the coworkers in addition to specialized tasks	3(3.8)	55(68.8)	22(27.5)
4	Control processes of medical and non-medical error control and prevention	6(7.5)	52(65)	22(27.5)
5	Talent, knowledge, and awareness in detection and prediction of the incidents	5(6.2)	56(70)	19(23.8)
6	Patient safety regulations and guidelines in the hospital and their value	2(2.5)	47(58.8)	31(38.8)
7	Foresight in the model of HROs	31(38.8)	38(47.5)	11(13.8)
8	Holding sessions with managers and staffs to present ways for error prevention	4(5)	56(70)	20(25)
9	Factors leading to irritation and discouragement of the managers and staffs	7(8.8)	52(65)	21(26.2)
10	How to prevent personal errors and mistakes	1(1.2)	46(57.5)	33(41.2)
11	Important factors in error prevention by implementation of HROs model	37(46.2)	39(48.8)	4(5)
12	Important factors in identification of HROs	48(60)	29(36.2)	3(3.8)
13	Informed relationship among staffs	39(48.8)	36(45)	5(6.2)
14	Avoiding simplification of analyses	47(58.8)	30(37.5)	3(3.8)
15	Reluctance to simplify interpretations	31(38.8)	44(55)	5(6.2)
16	Final objective of establishment of HROs model	40(50)	35(43.8)	5(6.2)
17	Outcome of respectful interactions of staff s	34(42.5)	32(40)	14(17.5)
18	How to analyze incidents and problems in the hospital	6(7.5)	60(75)	14(17.5)
19	How to receive feedback on self-activities the hospital	8(10)	55(68.8)	17(21.2)
20	How to access required resources to confront unexpected events	3(3.8)	55(68.8)	22(27.5)
21	How to gain experience from mistakes and errors of other staffs	2(2.5)	51(63.8)	27(33.8)
22	How to transfer critical information and make efforts to improve effective performance of organization	5(6.2)	57(71.2)	18(22.5)

Table 2 shows the differences in the frequency distribution of HROs dimensions observation in medical and non-medical departments.

**Table 2:** Relative distribution of HROs dimensions observation in Farabi eye hospital’s departments

***HROs dimensions***	***Observation level***	***Relative Observation (%)***		***Level of Significance***
		**Medical Department**	**Nonmedical Departments**	
Attention to patient safety	Not at all	-	-	0.663
	To some extent	59.1	63.9	
	Very much	40.9	36.1	
Hospital concerns about correcting errors	Not at all	-	-	0.653
	To some extent	70.5	75	
	Very much	29.5	25	
Reluctance to simplify interpretations by staff	Not at all	-	-	0.416
	To some extent	72.7	6	
	Very much	27.3	19.4	
Sensitivity to operation	Not at all	-	-	0.489
	To some extent	59.1	66.7	
	Very much	40.9	33.3	
Commitment to resilience	Not at all	-	-	0.003
	To some extent	44.7	6	
	Very much	52.3	19.4	
Deference to expertise	Not at all	-	-	0.004
	To some extent	52.3	83.3	
	Very much	47.7	16.7	

By Mann-Whitney test “Commitment to resilience” and “Deference to expertise” were significantly higher in medical versus non-medical departments (*P*=0.003, *P*=0.004).

Our results showed no significant correlation between the variable of knowledge of staffs and managers with HROs model establishment in Farabi Hospital (Table 3).

**Table 3:** Relationship between knowledge of staffs and managers with dimensions of HROs model establishment in Farabii Eye Hospital

***HROs dimensions***	***Knowledge of staff and managers***	
	**Spearman’s correlation coefficient**	**Level of significance**
Attention to patient safety	0.144	0.203
Hospital concerns about correcting errors	0.063	0.500
Reluctance to simplify interpretations by staff	−0.042	0.709
Sensitivity to operation	0.091	0.422
Commitment to resilience	−0.041	0.716
Deference to expertise	0.131	0.246

## Discussion

Since hospitals are one of the most important health care centers and receive the highest share of the health system resources, special attention should be paid to quality of the hospital services. The standards included in HROs model can be regarded as one of the most effective methods of guaranteeing and improving the quality of the hospital services (21).

If Farabi Eye Hospital wishes to be recognized as HROs model, it should pay more attention to increasing the quality of the services and improving the knowledge and learning status of its staff, and also managers should be accountable for possible problems and incidents. For this purpose, it is required to observe several important items and make the necessary. The hospital is a high-risk environment. High-reliability behaviors require the staffs and managers to have the capability of foreseeing the risks and problems and ways to confront them. To have an optimal performance is the other factors that can transform a hospital to HROs (29). In other words, high reliability hospitals should focus on identification of the errors and their reasons, have a teamwork attitude toward decision making, be aware of the impact of the decisions on hospital activities and performance, use problem-solving strategies creatively, and find the roots of errors and mistakes instead of reprimanding and criticizing the staffs (30).

Although the report of Landmark Medical Institute on human errors was published more than a decade ago, health care experts still continue their efforts to prevent patiently related risks. However, the question is, how can we make the health care system even more reliable? The answer is not only the staff’s hard work, but other factors also play a role in high reliability in the area of health care system (3). A comprehensive view in response to the above-mentioned question is to create the levels of safety and quality in the health care system, similar to other industries like aviation and nuclear energy. Health care experts, and policy makers in the public, and private sector have always tried to remove the problems related to safety and quality in these organizations. In addition, main standards of high-reliability organizations help the hospitals and other health care provider centers to improve the safety and quality of their services (31, 21).

Many health care centers have managed to allocate large amounts of money to high reliability; however, there are serious shortcomings in the safety and quality of the services provided by these centers (31). In fact, the risk of errors and mistakes, which may result in physical and mental damage to the patients, is on the rise in these organizations because the admitted patients with acute conditions require more complicated care (32). Hospital managers should continuously highlight the importance of safety, mutual trust among the staffs, and creating a culture of learning to correctly identify and analyze the occurrences and errors in order to use its results to improve the outcomes (33).

Different pressures threaten the pillars of the health care system in every country and test the limits of reliability; therefore, it is very important to observe civility and politeness combined with courtesy and kindness as very important issues in the safety culture of the hospitals (34). Moreover, the safety culture and comprehensive quality cannot be achieved with intimidating behaviors. Inappropriate behaviors hinder teamwork in the hospital and impede the delivery of quality services to the patients. Therefore, to achieve the standards of high-reliability organizations, the following steps should be taken properly in health care centers, especially hospitals:

1. Training all the staffs to observe politeness, courtesy and civility in all communications including telephone, face-to-face, etc.
2. Requiring the staffs as team member to present desirable behaviors as a standard model and observe ethical code fairly and continuously.
3. Removing risky behaviors in the organization
4. Supporting the staffs who report inappropriate behaviors
5. Emphasis on apology and soothing the families and patients faced inappropriate behaviors
6. Performing disciplinary actions in the organization with prior announcement
7. Developing a search and forward system for inappropriate behaviors and making use of interactive approaches to prevent intimidating behaviors (35).

One of the most important factors in becoming an HRO, considered by hospital managers, is to use systematic methods to analyze the causes of failure in health care services and to create purposeful solutions to complicated problems (36). On their path to becoming an HRO, hospitals should make every attempt to achieve magnificence and grandeur. For this reason, the first step is self-assessment of the current and ongoing status of the organization in terms of leadership, management, and safety culture, which provides the staff with comprehensive knowledge on the efforts of the organization for enhancement and improvement (37–39).

The insufficient similar studies at national and international levels, prolonged the time of assessment, using self-assessment for determination of managers and staffs’ knowledge about HROs model, and finally cross-sectional for this research are the limitation of this study.

## Conclusion

1. Priority should be given to achieving the standards of HROs and implementing its concepts in their organizational structure and culture
2. Commitment of the hospital managers and leaders to the model of HROs and their support in this regard are necessary.
3. Expanding and developing the safety culture, teamwork, considering an appropriate reward system (when the staffs discover an error which encourages them to report it), and creating an atmosphere of trust between the managers, staffs, and patients are of paramount importance.

## Ethical considerations

Ethical issues (Including plagiarism, informed consent, misconduct, data fabrication and/or falsification, double publication and/or submission, redundancy, etc.) have been completely observed by the authors.
